# Accelerometer-Measured Physical Activity and Sedentary Behavior Patterns in Taiwanese Adolescents

**DOI:** 10.3390/ijerph16224392

**Published:** 2019-11-10

**Authors:** Wen-Yi Wang, Yu-Ling Hsieh, Ming-Chun Hsueh, Yang Liu, Yung Liao

**Affiliations:** 1 Graduate Institute of Sports Pedagogy, University of Taipei, Taipei 11153, Taiwan; wenyiwang1225@gmail.com (W.-Y.W.); aoquan111@gmail.com (Y.-L.H.); 2 School of Physical Education and Sport Training, Shanghai University of Sport, Shanghai 200438, China; docliuyang@hotmail.com; 3 Shanghai Research Center for Physical Fitness and Health of Children and Adolescents, Shanghai University of Sport, Shanghai 200438, China; 4 Department of Health Promotion and Health Education, National Taiwan Normal University, Taipei 106, Taiwan; liaoyung@ntnu.edu.tw

**Keywords:** accelerometer, physical activity, sitting time, youth, sedentary behavior, teen health issues, school-based intervention

## Abstract

Levels of physical activity and sedentary behavior among adolescents seem to vary within different settings, but few Asian studies have compared physical activity and sedentary activity patterns in adolescents across weekdays/weekends and during-school time/after-school time. This study aimed to provide objectively measured data describing intensity-specific physical activity and sedentary behavior patterns in Taiwanese adolescents. The results were sorted by gender and divided between weekdays/weekends and during-school time/after-school time. A total of 470 Taiwanese students (49.6% boys, ages 12–15 y) were recruited and fitted with GT3X+ accelerometers for seven days. Intensity-specific physical activity, total sedentary time, and sedentary bouts (number and duration ≥30 min) were measured. The Mann-Whitney U test was used to examine the significant differences in physical activity and sedentary behavior patterns between the genders on weekdays/weekends and during school/after-school time. The results show that the adolescents’ overall activity levels were below recommended thresholds, with girls engaging in significantly less moderate to vigorous physical activity, having longer sedentary time, longer time spent in sedentary bouts, and more frequent sedentary bouts than boys. Similar results were observed in physical activities of each intensity as well as sedentary behavior variables, both on weekdays/weekends and during-school/after-school periods. These findings emphasize the importance of developing and implementing approaches to increase moderate to vigorous physical activity, as well as decrease prolonged sedentary time and long sedentary bouts, especially for Taiwanese girls.

## 1. Introduction

Physical inactivity is known to negatively affect both mental and physical health in adolescents [1]. The World Health Organization advocates that adolescents should engage in at least 60 minutes per day of moderate-to-vigorous physical activity (MVPA) [2]; nonetheless, MVPA levels in adolescents are commonly lower than 60 min per day [3]. For example, data indicate that a substantial majority of adolescents (approximately 80% of 13- to 15-year-olds) do not meet the physical activity guideline [4]. This means that roughly four out of every five adolescents globally are insufficiently physically active, although boys are more active, on average, than girls [5]. Similarly, in Taiwan, fewer than 20% of adolescents engage in the recommended 1 h of MVPA/day [6]. Consequently, it is crucial to describe the patterns of physical activity in adolescents in order to determine how this shortcoming can be addressed.

Previous studies indicate that subjective measurement may either underestimate or overestimate activity [7]. Compared to self-reports, objectively assessed tools such as accelerometers can provide the potential detail in patterns of daily physical activity [8]. For example, one traditional pattern of physical activity to emerge from self-reported measurements is that of the “weekend warrior” [9]. This may cause difficulties in measuring the pattern of physical activity and may not provide sufficiently detailed data for physical activity [10]. Therefore, using accelerometers allows us to examine the detailed physical activity patterns of adolescents across intensity-specific levels and different time periods. Moreover, in order to address physical inactivity observed in adolescents on weekends or after-school time on weekdays, it is beneficial to target physical activity interventions during these periods [11]. Nevertheless, little is known about intensity-specific physical activity across weekdays/weekends and during-school/after-school time [11].

According to the ecological model of health behavior [12], adolescents’ physical activity and sedentary behavior may occur in different environments, such as the home, at school, and in the community. Therefore, it is critical to better understand how adolescents engage in physical activity and sedentary behavior in both school and non-school environments using appropriate measurements. Although some studies that used accelerometers to explore intensity-specific physical activity among boys and girls on weekday/weekend days have been conducted in Western countries [13,14], few have been carried out in Asian countries [15]. Additionally, little research has been carried out to compare intensity-specific physical activity during the weekday/weekend and during-school/after-school time relating to gender. Furthermore, although boys and girls have been found to have different patterns of physical activity [13,14,15], these results were inconsistent between intensity-specific physical activity on weekends versus weekdays by gender. For example, Generelo et al. [13] found Spanish adolescent boys have higher levels of MVPA compared with girls during both weekdays and weekends, but no gender difference was found in light physical activity (LPA). In contrast, another study conducted in four European countries showed no gender difference in MVPA between weekdays and weekend days (15 years old) [14]. Among Japanese adolescents (aged 12–14 years), it has been reported that girls engage in each intensity of physical activity, including LPA, moderate physical activity (MPA), and vigorous physical activity (VPA), less often than boys on both weekdays and weekends (the only exception is LPA on weekends) [15]. However, no studies have yet enlisted accelerometers to objectively assess daily intensity-specific physical activity differences by gender in Taiwanese adolescents. Therefore, examining gender differences in LPA, MPA, VPA, and MVPA separately, based on different time periods, can help us to understand the current status of these different types of physical activity across genders and may be helpful in determining how to better design gender-specific interventions to increase physical activity in the future.

Sedentary behavior has been defined as seated or reclined posture with low level of metabolic equivalent tasks (<1.5 METs) during waking hours, as when one watches television, plays video games, or reads [16]. Time spent in sedentary behavior has been found to be an independent behavior factor for health risks in adolescents, such as metabolic disease, being overweight, and cardiovascular disease, and to have a negative impact on academic achievement, fitness, and psychological health [17]. The amount of sedentary time that correlates with health risks can accumulate even when meeting physical activity guidelines [18]. For example, several studies reported that both genders had levels of physical activity that were higher than average, while at the same time, they showed higher levels of screen time or social-related sedentary activity [18]. According to the Taiwan Youth Health Survey, participation in physical activity among Taiwanese adolescents for 60 min at least three times per week was reported in approximately 66% of boys and 52.6% of girls. However, the survey also reported excessive sedentary time (such as watching TV, using the computer, and playing video games): 70.9% for boys and 79.5% for girls [19]. In addition, another previous study used the International Physical Activity Questionnaire to estimate adolescents’ physical activity levels, and found that the rates of participation in low, moderate, and high physical activity were 41.7%, 38%, and 20.3% [20].

In addition, although most studies link total time in sedentary behavior with unhealthy outcomes in adolescents [16], there is a lack of evidence as to accurately explore how sedentary behavior is accumulated, that is, long bouts of uninterrupted sedentary time at different times throughout the day. This investigation is important because school settings appear to promote continuous periods of sedentary time. Experts in sedentary behavior evaluation suggest that in addition to total sedentary time, we should also consider other elements, such as how sedentary behavior is distributed in terms of sedentary bouts, when analyzing the data [21]. Specific trends in uninterrupted sedentary time in adolescents remain to be specified. Only a limited number of studies have addressed how sedentary patterns (with respect to bouts and durations) differ by gender across weekdays/weekends and during-school/after-school time. Thus, in order to fill the above-mentioned research gap and strengthen the evidence regarding physical activity in non-western settings, this study investigated gender differences by objectively assessing the intensity-specific physical activity and sedentary behavior patterns of Taiwanese junior high school adolescents across weekdays/weekends and during-school/after-school periods. We hypothesized that physical activity and sedentary behavior patterns in this adolescent population may differ by gender, and moreover, that these differences would be evident across weekdays/weekends and during-school/after-school periods.

## 2. Materials and Methods

### 2.1. Participants

We used convenience sampling to contact 61 junior high schools in Taipei City, first by email and then by letter, between October 2015 and January 2016 during the school year. After receiving information about the study, 57 schools were excluded because they were not confident that they would be able to finish the program; thus, four public schools joined the study. A total of 2119 Taiwanese junior high school adolescents (three grades, age range 12–15 years) from the four schools were asked to participate in this cross-sectional study. Leaflets were used to invite the adolescents to take part in the study conducted at their school, with only those who were physically disabled being excluded from the study. While 566 initially agreed to participate in the study, 96 participants were subsequently withdrawn due to revocation of the study agreement and/or missing data, leaving 470 adolescents (boys = 233 and girls = 237, mean age 14.0 ± 0.7 y) for which we had valid data in analyses. Physical activity and sedentary behavior data were collected from March 2016 to July 2017. Each participant and their parent(s) signed a written informed consent to participate our study, and the Ethical Committee of the University of Taipei institutional review board (IRB No. 2016001) approved the study protocol.

### 2.2. Measures

#### Physical Activity and Sedentary Behavior

Intensity-specific physical activity and sedentary behavior patterns were assessed by GT3X+ accelerometers (ActiGraph, Pensacola, FL, USA). The GT3X+ accelerometer was selected because it is the most commonly used accelerometer in studies of adolescent PA [22,23]. The GT3X+ model has a dynamic range of ±6 g and is initialized to collect data in 15-second epochs (at 30 Hz) [24,25]. Following the standardized protocol for the accelerometer [26], we asked the participants to wear the accelerometer on the right side of their waist, positioned above the right hip [24,25] for 7 consecutive days at all times except when sleeping or engaging in water-based activities such as swimming or showering. The accelerometer data were considered valid if at least 4 valid days (3 weekdays + 1 weekend day) and at least 600 min in a day were recorded [25]. Non-wearing of the accelerometer was defined as a period of at least 60 consecutive minutes of no activity, allowing for two consecutive minutes of observations between 1 and 100 counts [27]. ActiGraph data were downloaded using ActiLife version 6.11.4 (ActiGraph, Pensacola, FL, USA) and saved in raw format as GT3X files.

According to previous studies on adolescents [22,23], each activity variable was defined using a cut point of ≤100 counts/min to indicate sedentary behavior, 101–2295 counts/min to indicate LPA, 2296–4011 counts/min to indicate MPA, and ≥4012 counts/min to indicate VPA, while MPA and VPA were combined to calculate MVPA. These cut-offs values were subsequently validated for adolescents [28]. All intensity-specific physical activity time variables were reported in min/day. Daily total sedentary time was calculated by summing each 15-s epoch the cut-point of less than 100 counts/min [27], which is also validated for adolescents [28]. Sedentary bouts were defined as a period of consecutive minutes at least ≥30 min where the accelerometer registers < 100 counts/min [29]. Two sedentary bouts variables were classified as: (1) total time in duration of sedentary bouts (at least ≥30 min) and (2) number of sedentary bouts. An average for all valid days was calculated for each summary measure. Wearing time was calculated in min/day. All variables were separated into “7 days” (from Monday to Sunday), “weekdays” (from Monday to Friday), “weekend” (Saturday and Sunday), “during-school time” (from Monday to Friday 07:20 to 17:00), and “after-school time” (from Monday to Friday 17:00 until going to sleep).

### 2.3. Statistical Analyses

In the analysis, the Kolmogorov-Smirnov test revealed that the data regarding each variable of physical activity and sedentary behavior patterns were not normally distributed (*p* < 0.05). Because the distribution of the data was skewed, the Mann-Whitney U test was used to examine the significant differences in the means (standard deviations [SDs]) of the intensity-specific physical activity and sedentary behavior patterns between the genders on weekdays/weekends and during school/after-school time on weekdays. We used ActiLife version 6.11.4 (ActiGraph, Pensacola, FL, USA) data analysis software and IBM SPSS 23.0 (Inc, Chicago, IL, USA) for all the statistical analyses. The significance level was set at *p* < 0.05.

## 3. Results

### 3.1. Description of Study Participants

Table 1 shows the demographic variables of the 470 study participants who provided valid data for analysis. The gender distribution was almost equal among the participants (49.6% were boys). The means of the participants’ age were 14.0 ± 0.7 years. The mean ± SD of accelerometer wear time was 14.4 ± 2.0 h.

### 3.2. Total Amounts and Patterns of Physical Activity and Sedentary Behavior in 7-Day Period

Table 2 shows the intensity-specific physical activity and sedentary behavior patterns over a 7-day period. Overall, time spent in LPA, MPA, VPA, and MVPA was 230.5 (±68.8), 21.9 (±12.6), 6.9 (±7.5) and 28.7 (±18.6) minutes/day, respectively. Furthermore, total sedentary time and duration of sedentary bouts were 10.1 (±1.9) and 5.1 (±2.1) hours/day, respectively. The daily number of sedentary bouts was 6.1 (±2.3).

The statistical analyses of the differences between genders in relation to each intensity level of physical activity and sedentary behavior patterns revealed that girls spent significantly less time in LPA, MPA, VPA, and MVPA within a 7-day period compared to boys. Regarding sedentary behavior, the results showed that, over a 7-day period, girls had significantly higher total sedentary time, which included more and longer sedentary bouts compared to boys.

### 3.3. Total Amounts and Patterns of Physical Activity and Sedentary Behavior on Weekdays and Weekends

Table 3 shows the intensity-specific physical activity and sedentary behavior patterns on weekdays/weekends. On weekdays, compared with boys, girls spent significantly less time in daily MPA, VPA, and MVPA, and had more total sedentary time. Girls also had sedentary bouts of a higher number longer duration than boys on weekdays. During the weekend, compared with boys, girls spent significantly less time in daily MPA, VPA, and MVPA. However, no significant gender differences were observed in time spent on LPA, total sedentary time, duration of sedentary bouts, or number of sedentary bouts during the weekend.

### 3.4. Total Amounts and Patterns of Physical Activity and Sedentary Behavior during School and after School

Table 4 shows the intensity-specific physical activity and sedentary behavior patterns during and after school. The data show that boys spent significantly more time in LPA, MPA, VPA, and MVPA compared with girls during-school time. Moreover, girls spent more total sedentary time, had sedentary bouts of longer duration, and had a higher number of sedentary bouts during school time. The results also show that girls engaged in less MPA, VPA, and MVPA than boys after school time. In relation to sedentary behavior patterns, girls engaged in longer total sedentary time, sedentary bouts and had higher numbers of sedentary bouts than boys after school time. However, the time spent in LPA showed no significant difference between genders in the after-school time periods.

## 4. Discussion

This study is the first to employ accelerometers for the analysis of intensity-specific physical activity and sedentary behavior patterns in Taiwanese adolescents, with separate weekday/weekend and during-school/after-school contexts. Overall, we found that Taiwanese adolescents engaged in insufficient daily MVPA (28.7 min/day) compared to the guideline recommendations for physical activity [2]. We also found that Taiwanese adolescents spent considerable time engaging in sedentary behavior (10.1 h/day) in comparison with those in Japan [15,30], Singapore [31], and Western countries [13,14]. Moreover, these results were obtained using objective methods that provided detailed and specific data compared to previous studies conducted in Taiwan that used subjective methodologies [5]. The findings highlight the urgent need to develop strategies to promote greater MVPA, limit prolonged sedentary time, and modify sedentary behavior patterns among Taiwanese adolescents in order to obtain health benefits.

### 4.1. Patterns of Physical Activity and Sedentary Behavior by Gender

Our results show that there are gender differences in the patterns of physical activity and sedentary behavior, and we observed that adolescent boys are more active in MVPA and spend less time being sedentary than girls. The results are consistent with previous findings that used objective measurements to analyze MVPA [32] and total sedentary time [15,30,31] in adolescents. These results might be explained in part by biological factors [33]. For example, the strength per gram of muscle becomes greater in boys in adolescence because of variance in the biochemical nature and structure of the muscle cells as induced by male sex hormones [34]. Another reason for the gender differences could be the effects of a socio-cultural environment [35], where adolescent girls’ participation in physical activity is influenced by social subjective norms [36]. The norm makes it more likely for boys to meet screen time recommendations (e.g., TV time < 2 h/day) if parents prefer their male children to do physical activities but do not encourage girls to do so [37]. This could lead to girls more easily alternating between physical activities and sedentary behaviors [38]. However, because these data were collected by accelerometers, it does not indicate what the adolescents were doing during sedentary periods. In addition, while our results also add to previous findings by revealing that the adolescent girls engaged in more uninterrupted sedentary bouts than the boys, the reason or reasons for this difference cannot be explained using the data gathered in this study. Thus, whether these results reflect actual gender differences among Taiwanese adolescents in terms of MVPA and sedentary behaviors and whether such differences are the result of ecological characteristics should be considered in future research. Moreover, future studies should utilize both accelerometers and self-reports concurrently in order to obtain more comprehensive assessments of sedentary behavior patterns in boys and girls.

### 4.2. Physical Activity and Sedentary Behavior Patterns on Weekdays/Weekends and during-School Time/After-School Time by Gender

According to the ecological model of health behavior [12], adolescents’ physical activity and sedentary behavior may occur in different environments, such as school and non-school settings. Our results showed that for the weekday/weekend and during-school/after-school time, girls had less MVPA time and more total sedentary time than boys, except for total sedentary time on weekends. This strengthens our notion that these results point to the need for the development of tailored interventions for different settings (i.e., during-school, after-school tutoring, home). This is consistent with a previous study [39]. Our results also showed that for the weekday/weekend and during-school/after-school time, girls had less MVPA time and more total sedentary time than boys, except for total sedentary time on weekends. This strengthens our notion that these results point to the need for the development of comprehensive interventions for different settings (weekdays/weekends and during-school/after-school tutoring, home). When designing intervention programs to promote MVPA, there should be a focus on both weekdays and weekends [40,41], especially for adolescent girls. Additionally, strategies that aim to reduce sedentary behavior should focus on intervention during weekdays both during [42] and after school [43] priority for adolescent girls. In Taiwan, there are limited opportunities for extra-curricular physical activities either during the week or on weekends. Moreover, these activities usually focus on competitive sports (such as baseball or basketball), and therefore, engage only a minority of adolescent boys. Besides focusing on the promotion of school physical activity, greater efforts to intervene during weekends, such as in the family or community setting, seems to be important [44], and adolescent girls should be prioritized in these interventions. Moreover, the results showed no gender difference in total time spent in sedentary behavior on weekends. Consequently, these results suggest that even though MVPA remains relatively higher in boys than in girls on weekends, there is a need for developing gender-specific intervention programs on weekends for both boys and girls.

### 4.3. Strengths and Limitations

The results add to previous findings by revealing that adolescent girls have more uninterrupted sedentary bouts across weekdays as well as during and after school (except on weekends) when compared to boys. Several studies have supported the notion that both the duration and the sedentary bouts frequency are associated with risk of cardio-metabolic disease [45], waist circumference [46], and all-cause mortality [47]. The evidence indicated that the patterns of sedentary behavior in adolescence will carry over into adulthood lifestyle over time [48]. Therefore, public health programs should take account of the gender-specific nature of the correlates of adolescents’ sedentary behavior patterns.

This study has several limitations. First, students from only four schools took part in the study, which restricted the scope of the results, such that our sample cannot be considered representative of a larger population. Second, since the study participants were selected from metropolitan areas, our results may not apply to students in other regions of Taiwan. Third, the environments we examined were limited only to school environments and non-school environments in general. Specific non-school environment domains, such as transport, home, and community settings, were not interpreted in our studies. Four, the during-school and after-school times were defined from Monday to Friday from 7:20 to 17:00 and from 17:00 until going to sleep, respectively. This school time setting is different from other countries, such as Singapore (7:00 to 15:00) [31] and the UK (9:00 to 15:30) [49]. In Taiwan, students typically attend school from 7:00 a.m. to 5:30 p.m., with minimal variation in this timeframe. Therefore, our results correlating to the during-school and after-school times are difficult to compare with other countries’ settings. However, these findings may also highlight that the extended school hours that are common in many Asian countries may be of significance in relation to the frequency and duration of sedentary behaviors, and therefore, to the need for interventions in school or outside of school. On the other hand, despite the use of the GT3X+ accelerometers in this study having been carried out according to appropriate protocols, the accelerometers were not used 24 h per day. As such, the data collected in this study might have underestimated the PA levels and sedentary time of the students due to the removal of the accelerometers during sleep and water-based activities such as swimming in physical education class. Moreover, unlike data provided by other devices (e.g., activPAL™ activity monitor), the GT3X accelerometer data are limited in that they cannot provide postural information (i.e., sitting vs. standing still), such that data from the accelerometer may overestimate sedentary time [50]. Finally, the absence of other information on the contexts and settings where both physical activity and sedentary behavior occur (such as weather conditions, geographic location, etc.) is one more limitation that should be considered.

## 5. Conclusions

This study identified that girls accumulated a lower MVPA, spent more time in sedentary behavior, and had longer and more frequent sedentary bouts than boys in a 7-day period, on weekdays, and during-school/after-school time (thus, nearly always except on weekends). The results emphasize the need to prioritize the development of intervention strategies to address the issue of insufficient physical activity engagement, and that particular attention should be given to adolescent girls’ MVPA on both weekdays and weekends. Moreover, when developing interventions that target a reduction in sedentary behavior, attention should be paid to adolescent girls’ total sedentary time, as well as the duration and frequency of sedentary bouts on weekdays during school and after school.

## Figures and Tables

**Table 1:** Demographic characteristics of study participants.

Variables	Total Sample (*n* = 470)	Boys (*n* = 233)	Girls (*n* = 237)
	M ± SD	M ± SD	M ± SD
Age (years)	14.0 ± 0.7	14.0 ± 0.7	14.1 ± 0.7
Weight (kg)	53.6 ± 13.3	57.4 ± 15.4	50.0 ± 9.7
Height (cm)	160.9 ± 7.6	164.3 ± 7.8	157.6 ± 5.7
Accelerometer wearing time (in hours)	14.4 ± 2.0	14.2 ± 2.2	14.6 ± 1.9

M = mean; SD = standard deviation.

**Table 2:** Time spent in objectively measured PA and SB patterns in adolescents in a 7-day period.

Variables	Total Sample (*n* = 470)		Boys (*n* = 233)	Girls (*n* = 237)	*p*
LPA, minutes/day	M ± SD	230.5 ± 68.8	240.8 ± 73.9	220.3 ± 61.8	0.004 *
	Median	223.6	232.6	214.9	
	IQR	(185.3, 268.9)	(189.0, 284.4)	(181.0, 255.4)	
MPA, minutes/day	M ± SD	21.9 ± 12.6	26.6 ± 13.4	17.2 ± 9.9	<0.001 **
	Median	20.4	25.8	15.6	
	IQR	(12.7, 28.8)	(17.8, 33.3)	(9.6, 22.4)	
VPA, minutes/day	M ± SD	6.9 ± 7.5	9.8 ± 8.6	4.0 ± 4.5	<0.001 **
	Median	4.2	7.7	2.8	
	IQR	(2.1, 9.0)	(3.4, 13.3)	(1.5, 4.8)	
MVPA, minutes/day	M ± SD	28.7 ± 18.6	36.4 ± 20.4	21.2 ± 12.6	<0.001 **
	Median	25.5	34.2	18.6	
	IQR	(15.2, 37.8)	(21.8, 46.8)	(12.8, 27.0)	
Total sedentary time, hours/day	M ± SD	10.1 ± 1.9	9.5 ± 2.0	10.6 ± 1.7	<0.001 **
	Median	10.3	9.6	10.7	
	IQR	(9.0, 11.4)	(8.5±10.9)	(9.7, 11.7)	
Duration of sedentary bouts, hours/day	M ± SD	5.1 ± 2.1	4.5 ± 2.2	5.7 ± 1.9	<0.001 **
	Median	5.2	4.3	5.7	
	IQR	(3.5, 6.7)	(2.8, 6.0)	(4.4, 7.2)	
Number of sedentary bouts	M ± SD	6.1 ± 2.3	5.5 ± 2.4	6.8 ± 2.0	<0.001 **
	Median	6.3	5.4	6.8	
	IQR	(4.5, 7.7)	(3.5, 7.3)	(5., 8.0)	

Significant difference * (*p* < 0.05), ** (*p* < 0.001). PA = physical activity; SB = sedentary bout; LPA = light physical activity; MPA = moderate physical activity; VPA = vigorous physical activity; MVPA = moderate-to-vigorous physical activity; M = mean; SD = standard deviation; IQR = interquartile range.

**Table 3:** Time spent in objectively measured PA and SB patterns for adolescents on weekdays and weekends.

Variables		Weekday		*p*	Weekend		*p*
		Boys (*n* = 233)	Girls (*n* = 237)		Boys (*n* = 233)	Girls (*n* = 237)	
LPA, minutes/day	M ± SD	226.1 ± 86.0	209.3 ± 72.0	0.047 *	137.6 ± 114.6	131.7 ± 105.0	0.662
	Median	224.0	206.2		136.0	130.5	
	IQR	(164.6, 276.4)	(160.7, 255.5)		(0.0, 215.0)	(36.0, 196.5)	
MPA, minutes/day	M ± SD	25.9 ± 14.2	17.2 ± 9.8	<0.001 **	10.6 ± 15.0	6.5 ± 8.6	0.040 *
	Median	23.8	15.8		4.5	3.5	
	IQR	(16.4, 33.0)	(9.2, 23.7)		(0.0, 13.8)	(0.0, 10.5)	
VPA, minutes/day	M ± SD	9.4 ± 8.0	4.1 ± 4.9	<0.001 **	4.2 ± 10.1	1.3 ± 3.3	<0.001 **
	Median	7.2	2.6		0.5	0.0	
	IQR	(3.3, 13.5)	(1.6, 5.4)		(0.0, 3.3)	(0.0, 1.0)	
MVPA, minutes/day	M ± SD	35.3 ± 20.6	21.2 ± 13.0	<0.001 **	14.8 ± 23.7	7.8 ± 10.7	0.030 *
	Median	33.2	19.2		5.5	4.0	
	IQR	(20.5, 46.8)	(11.6, 28.7)		(0.0, 17.8)	(0.0, 11.0)	
Total sedentary time, hours/day	M ± SD	8.7 ± 2.5	10.0 ± 2.4	<0.001 **	5.9 ± 4.5	6.2 ± 4.3	0.279
	Median	8.9	10.3		6.1	6.6	
	IQR	(6.8, 10.5)	(8.3, 11.8)		(0.0, 10.0)	(2.8, 9.7)	
Duration of sedentary bouts, hours/day	M ± SD	4.1 ± 2.1	5.4 ± 2.0	<0.001 **	2.6 ± 2.6	2.9 ± 2.5	0.091
	Median	3.7	5.5		2.0	2.7	
	IQR	(2.6,5.4)	(3.8, 6.8)		(0.0, 4.2)	(0.3, 4.6)	
Number of sedentary bouts	M ± SD	5.1 ± 2.5	6.5 ± 2.4	<0.001 **	2.9 ± 2.8	3.2 ± 2.7	0.138
	Median	4.6	6.4		2.5	3.0	
	IQR	(3.4, 7.0)	(4.8, 8.2)		(0.0, 5.0)	(0.5, 5.5)	

Significant difference * (*p* < 0.05), ** (*p* < 0.001). PA = physical activity; SB = sedentary bout; LPA = light physical activity; MPA = moderate physical activity; VPA = vigorous physical activity; MVPA = moderate to vigorous physical activity; M = mean; SD = standard deviation; IQR = interquartile range.

**Table 4:** Time spent in objectively measured PA and SB patterns in adolescents during-school and after-school.

Variables		During-School		*p*	After-School		*p*
		Boys (*n* = 233)	Girls (*n* = 237)		Boys (*n* = 233)	Girls (*n* = 237)	
LPA, minutes/day	M ± SD	146.1 ± 56.0	130.2 ± 44.7	0.002 *	87.5 ± 76.0	78.2 ± 56.2	0.206
	Median	138.8	128.4		74.6	67.9	
	IQR	(106.4, 179.8)	(102.5, 155.8)		(49.7,101.6)	(45.6, 95.7)	
MPA, minutes/day	M ± SD	17.0 ± 10.5	10.6 ± 6.5	<0.001 **	9.7 ± 11.3	5.8 ± 6.1	<0.001 **
	Median	15.0	9.8		6.6	4.2	
	IQR	(9.4, 21.6)	(5.8, 14.0)		(2.4, 12.1)	(1.6, 7.5)	
VPA, minutes/day	M ± SD	6.4 ± 5.4	2.8 ± 2.6	<0.001 **	3.4 ± 6.6	1.4 ± 4.4	<0.001 **
	Median	4.4	2.0		1.2	0.4	
	IQR	(2.4, 9.2)	(1.0, 3.6)		(0.2, 4.1)	(0.0, 1.4)	
MVPA, minutes/day	M ± SD	23.5 ± 15.0	13.4 ± 8.2	<0.001 **	13.2 ± 17.1	7.2 ± 9.1	<0.001 **
	Median	20.8	12.3		8.6	4.8	
	IQR	(12.1, 31.2)	(7.4, 17.9)		(2.9, 15.3)	(2.0, 9.2)	
Total sedentary time, hours/day	M ± SD	5.3 ± 1.4	6.1 ± 1.2	<0.001 **	3.7 ± 2.5	4.1 ± 2.3	0.009 *
	Median	5.5	6.2		3.5	3.9	
	IQR	(4.2, 6.4)	(5.4, 7.0)		(2.2, 4.4)	(2.8, 4.8)	
Duration of sedentary bouts, hours/day	M ± SD	2.5 ± 1.5	3.4 ± 1.4	<0.001 **	1.6 ± 1.4	2.0 ± 1.3	<0.001 **
	Median	2.3	3.3		1.3	1.7	
	IQR	(1.4, 3.5)	(2.3, 4.4)		(0.7, 2.0)	(1.1, 2.4)	
Number of sedentary bouts	M ± SD	3.3 ± 1.8	4.4 ± 1.7	<0.001 **	1.8 ± 1.6	2.2 ± 1.6	<0.001 **
	Median	3.0	4.4		1.6	2.0	
	IQR	(1.9, 4.6)	(3.2, 5.6)		(1.0, 2.3)	(1.2, 2.8)	

Significant difference * (*p* < 0.05), ** (*p* < 0.001). PA = physical activity; SB = sedentary bout; LPA = light physical activity; MPA = moderate physical activity; VPA = vigorous physical activity; MVPA = moderate to vigorous physical activity; M = mean; SD = standard deviation; IQR = interquartile range.
