# Erratum: Wang, W.Y., et al. Accelerometer-Measured Physical Activity and Sedentary Behavior Patterns in Taiwanese Adolescents. *Int. J. Environ. Res. Public Health* **2019**, **16**, 4392

**DOI:** 10.3390/ijerph17249294

**Published:** 2020-12-11

**Authors:** Wen-Yi Wang, Yu-Ling Hsieh, Ming-Chun Hsueh, Yang Liu, Yung Liao

**Affiliations:** 1 Graduate Institute of Sport Pedagogy, University of Taipei, Taipei 11153, Taiwan; wenyiwang1225@gmail.com (W.-Y.W.); aoquan111@gmail.com (Y.-L.H.); 2 School of Physical Education and Sport Training, Shanghai University of Sport, Shanghai 200438, China; docliuyang@hotmail.com; 3 Shanghai Research Center for Physical Fitness and Health of Children and Adolescents, Shanghai University of Sport, Shanghai 200438, China; 4 Department of Health Promotion and Health Education, National Taiwan Normal University, Taipei 106, Taiwan; liaoyung@ntnu.edu.tw

Due to an error during production, Section 3.1 and Section 3.2 in the results section of the published paper [1] displayed incorrect data—corrected versions of the two sections are provided below. In Section 3.1, we found that Taiwanese adolescents’ total accelerometer wear time in the study was 11.72 h per day. Moreover, in Section 3.2, we found that Taiwanese adolescents’ total light physical activity (LPA), moderate physical activity (MPA), vigorous physical activity (VPA), and moderate-to-vigorous physical activity (MVPA) time in the study was 188.1 (±75.0), 17.4 (±10.7), 5.4 (±6.2), and 22.8 (±15.7) min/day, respectively. Regarding the sedentary patterns, the total sedentary time was 8.2 (±2.7) h per day, the duration of sedentary bouts was 4.1 (±2.0) h per day, and the number of sedentary bouts was 5.0 (±2.3). The authors would like to apologize for any inconvenience caused to the readers by this error.

## 3. Results

### 3.1. Description of Study Participants

Table 1 shows that the mean ± SD of accelerometer wear time was 11.72 (±3.47) h/day.

### 3.2. Total Amounts and Patterns of Physical Activity and Sedentary Behavior in 7-Day Period

Table 2 shows the intensity-specific physical activity and sedentary behavior patterns over a 7-day period. Overall, time spent in LPA, MPA, VPA, and MVPA was 188.1 (±75.0), 17.4 (±10.7), 5.4 (±6.2), and 22.8 (±15.7) min/day, respectively. Furthermore, the total sedentary time and duration of sedentary bouts were 8.2 (±2.7) and 4.1 (±2.0) h/day, respectively. The daily number of sedentary bouts was 5.0 (±2.3).

The statistical analyses of the differences between genders in relation to each intensity level of physical activity and sedentary behavior patterns revealed that girls spent significantly less time in MPA, VPA, and MVPA within a 7-day period compared to boys, but not LPA. Regarding sedentary behavior, the results showed that over a 7-day period, girls had significantly higher total sedentary times, which included more frequent and longer times in sedentary bouts compared to boys.

## 4. Discussion

This study is the first to employ accelerometers for the analysis of intensity-specific physical activity and sedentary behavior patterns in Taiwanese adolescents, with separate weekday/weekend and during-school/after-school contexts. Overall, we found that Taiwanese adolescents engaged in insufficient daily MVPA (22.8 min/day) compared to the guideline recommendations for physical activity. We also established that Taiwanese adolescents spent excessive time engaging in sedentary behavior (8.2 h/day) in comparison to those in Japan [15,30], Singapore [31], and Western countries [13,14].

## Figures and Tables

**Table 1:** Demographic characteristics of study participants.

Variables	Total Sample (*n* = 470)	Boys (*n* = 233)	Girls (*n* = 237)
	M ± SD	M ± SD	M ± SD
Age (years)	14.0 ± 0.7	14.0 ± 0.7	14.1 ± 0.7
Weight (kg)	53.6 ± 13.3	57.4 ± 15.4	50.0 ± 9.7
Height (cm)	160.9 ± 7.6	164.3 ± 7.8	157.6 ± 5.7
Accelerometer wearing time (in hours)	11.72 ± 3.47	11.43 ± 3.58	12.02 ± 3.32

M = mean; SD = standard deviation.

**Table 2:** Time spent in objectively measured PA and SB patterns in adolescents in a 7-day period.

Variables	Total Sample (*n* = 470)		Boys (*n* = 233)	Girls (*n* = 237)	*p*
LPA, minutes/day	M ± SD	188.1 ± 75.0	194.6 ± 81.8	181.6 ± 67.1	0.061
	Median	180.8	180.7	180.9	
	IQR	(135.7, 230.4)	(136.1, 241.0)	(135.2, 221.3)	
MPA, minutes/day	M ± SD	17.4 ± 10.7	20.8 ± 11.5	13.9 ± 8.5	<0.001 **
	Median	15.7	18.6	13.0	
	IQR	(9.7, 22.3)	(13.2, 27.0)	(7.3, 18.6)	
VPA, minutes/day	M ± SD	5.4 ± 6.2	7.7 ± 7.1	3.2 ± 4.1	<0.001 **
	Median	3.3	5.6	2.1	
	IQR	(1.7, 6.9)	(2.7, 10.7)	(1.1, 4.0)	
MVPA, minutes/day	M ± SD	22.8 ± 15.7	28.5 ± 17.4	17.2 ± 11.2	<0.001 **
	Median	19.7	25.3	15.6	
	IQR	(11.8, 29.2)	(16.6, 36.2)	(9.2, 23.0)	
Total sedentary time, hours/day	M ± SD	8.2 ± 2.7	7.7 ± 2.7	8.7±2.6	<0.001 *
	Median	8.2	7.5	8.7	
	IQR	(6.0, 10.3)	(5.4, 9.5)	(6.7, 10.7)	
Duration of sedentary bouts, hours/day	M ± SD	4.1 ± 2.0	3.6 ± 2.0	4.7 ± 1.9	<0.001 **
	Median	3.8	3.2	4.7	
	IQR	(2.5, 5.4)	(2.1, 4.7)	(3.2, 6.0)	
Number of sedentary bouts	M ± SD	5.0 ± 2.3	4.4 ± 2.3	5.6 ± 2.2	<0.001 **
	Median	4.7	4.0	5.4	
	IQR	(3.1, 6.6)	(2.7, 5.6)	(3.9, 7.1)	

Significant difference * (*p* < 0.05), ** (*p* < 0.001). PA = physical activity; SB = sedentary bout; LPA = light physical activity; MPA = moderate physical activity; VPA = vigorous physical activity; MVPA = moderate-to-vigorous physical activity; M = mean; SD = standard deviation; IQR = interquartile range.
